# The Influence of Cardiovascular Risk Factors and Hypogonadism on Cardiac Outcomes in an Aging Population of Beta-Thalassemia Patients

**DOI:** 10.3390/jcdd9010003

**Published:** 2021-12-21

**Authors:** Umberto Barbero, Matteo Ajassa, Carmen Maria Gaglioti, Antonio Piga, Giovanni Battista Ferrero, Filomena Longo

**Affiliations:** 1 Cardiology Unit, “Santissima Annunziata” Hospital, 12038 Savigliano, Italy; 2 Reference Centre for Haemoglobinopathies, Department of Clinical and Biological Sciences, University of Turin, 10043 Turin, Italy; matteo.ajassa@gmail.com (M.A.); carmenmaria.gaglioti@unito.it (C.M.G.); antonio.piga@gmail.com (A.P.); giovannibattista.ferrero@unito.it (G.B.F.); filomena.longo@unito.it (F.L.)

**Keywords:** thalassemia, cardiovascular risk, iron related disease, hypogonadism

## Abstract

Beta-thalassemia major (β-TM) is a hereditary genetic disease worsened by many comorbidities due to transfusion-related iron despite chelation therapy. Since there has recently been an increase in life expectancy of patients to up to 50 years old, which influences the prevalence of these diseases and the time span for traditional cardiovascular risk factors to play their role, this study aims to evaluate their distribution and prevalence in a population of thalassemia major patients and their relationship with observed cardiovascular events and potential modifying factors. One hundred and fifty-nine β-TM patients with at least 15 years of follow-up were included in this study. The mean age was 40.9 ± 8.4 years; 28% had diabetes mellitus and 62% had hypogonadism. The cardiovascular risk assessed using algorithms (CUORE and Pooled Cohort Risk Equation—PCRE) was low, but 3.8% of patients had at least one episode of heart failure, 35.9% showed early signs of heart failure, 22% received a diagnosis of diastolic dysfunction, and 21.4% showed supraventricular arrhythmias. Hypogonadism was shown to be related to the occurrence of cardiovascular events. The chronic accumulation of iron in the heart and the specific metabolic profile, mainly observed in patients with hypogonadism, allows us to define β-TM as a condition with a high level of cardiovascular risk from many points of view (iron-related myopathy, atherosclerosis and arrhythmias), which requires better stratification tools and a specific follow-up program.

## 1. Introduction

Beta-thalassemia major (β-TM) is a hereditary hemoglobin (Hb) disorder caused by reduced synthesis of the β-globin chain, resulting in a chronic hemolytic anemia that typically requires lifelong transfusion therapy [1]. These patients develop severe anemia and bone marrow hyperplasia, leading to jaundice, leg ulcers, cholelithiasis, massive splenomegaly, the thickening of the cranial, malar bones, and long bones, pathologic fractures, and impaired growth. β-TM natural history is characterized by a bad prognosis in the first decade of life. Frequent transfusions lead to iron overload and organ damage: glucose intolerance, diabetes mellitus, skin hyperpigmentation, hypothyroidism, hypogonadism, liver hepatitis, and cirrhosis; finally, cardiomyopathy develops with heart failure, which still is the leading cause of death if iron chelation is not adequately performed [1,2].

Thanks to chelation therapy, the life expectancy of these patients has increased from 16 years in 1964 to 50 years today, with a marked improvement in the quality of life [3,4]. Despite the continued presence of iron-related heart disease as a main concern, increasing life expectancy has carried those patients into an age span in which the extremely common atherosclerotic disease may begin to threaten them [5,6,7]. Cardiac disease still remains a major concern, with many efforts taking place to explore early cardiac dysfunction [8,9]. Furthermore, increased inflammation due to iron-overload pathophysiology could accelerate the atherosclerotic process, while juvenile diabetes and endocrine alterations may set in earlier than in non-thalassemic people [10,11,12]. In order to assess the cardiovascular risk of beta-thalassemic patients, we evaluated the distribution and prevalence of cardiovascular risk factors and their relationship with observed cardiovascular events and endocrine status.

## 2. Materials and Methods

We screened a cohort of transfusion-dependent β-TM patients regularly followed up at Piedmont regional center for hemoglobinopathies of San Luigi Gonzaga hospital in Orbassano (Turin). Informed consent was obtained from all subjects involved in the study.

Inclusion criteria consisted of: a clinical and molecular diagnosis of β-thalassemia major, age of more than 18 years old, and at least fifteen years of follow-up at enrollment. Clinical and laboratory data were collected from the center’s electronic medical records, including the WebThal^®^ database.

Cardiovascular outcomes of interest were heart failure hospitalization, early clinical signs of heart failure (defined as signs or symptoms recorded as secondary to heart involvement and leading to a change in therapy such as the introduction of beta-blockers or diuretics), arrhythmias, first diagnosis of diastolic dysfunction, first diagnosis of ejection fraction lower than 55% (cut-off chosen based on previous guidelines on echocardiographic assessment of left ventricular systolic function [13], upon which the majority of older echocardiography was reported, and previous studies on heart failure [14]), myocardial infarction, stable and unstable angina, PTCA (Percutaneous Transluminal Coronary Angioplasty), and CABG (Coronary Artery Bypass Grafting).

Follow-up visits were scheduled every year alongside execution of echocardiography, electrocardiograms, and CMR with T2*. Blood samples for evaluations of patients’ iron statuses were collected before every transfusion day.

The predicted cardiovascular risk in the next ten years was evaluated by means of two established scores: firstly, the CUORE123 assessment by the Italian Istituto Superiore di Sanità (ISS) and secondly the atherosclerotic cardiovascular disease (ASCVD) risk with the Pool Cohort Risk Equation method124 (PCRE) by the American Heart Association (AHA). An accurate analysis of all available clinical data was performed and the endpoints were diagnosed as per methods defined by two researchers (M.A. and U.B.), and controversies were solved with inclusion of a third opinion (F.L.).

Comorbidities were diagnosed using the following criteria: hypothyroidism was diagnosed by measurement of blood TSH >4.0/mU/L with a low fT4 level; hypoparathyroidism was diagnosed upon identification of characteristic symptoms and abnormal levels of calcium, phosphorus, magnesium, creatinine, and intact parathyroid hormone; hypogonadism was diagnosed by measurement of blood concentration of LH, FSH, and Testosterone (in men) and Estrogen (in women); diabetes Mellitus was diagnosed by measurement of blood’s glucose levels and HBA1c concentration according to standardized ADA criteria (i.e., fasting plasma glucose ≥ 126 mg/dL (≥7.0 mmol/L), glucose plasma levels after Oral Glucose Tolerance Test ≥ 200 mg/dL (≥11.1 mmol/L), and HbA1C (≥6.5%).

Statistical methods: Categorical variables are reported as counts and percentages, whereas continuous variables as means and standard deviations or interquartile ranges (IQR). Whether to use a Gaussian or a non-Gaussian distribution was evaluated by the Kolmogorov-Smirnoff test. The *t*-test or the 3-way ANOVA were used to assess differences between parametric continuous variables, a Mann–Whitney U test was used for nonparametric variables, a chi-square test for categorical variables, and a Fisher exact test for 2 × 2 tables.

We constructed Kaplan–Meier cumulative-event curves in two groups with and without the intended features to describe the frequency of cardiac events according to time. The two curves were compared using the log-rank test. A Cox multivariate analysis was performed to assess the independent predictors of cardiac events, defined as every variable, which resulted in a difference with *p* < 0.1 in the univariate analysis. A two-sided *p*-value < 0.05 was considered statistically significant. All analyses were performed with SPSS 21.0 (IBM, Armonk, NY, USA).

## 3. Results

**Figure 1:**
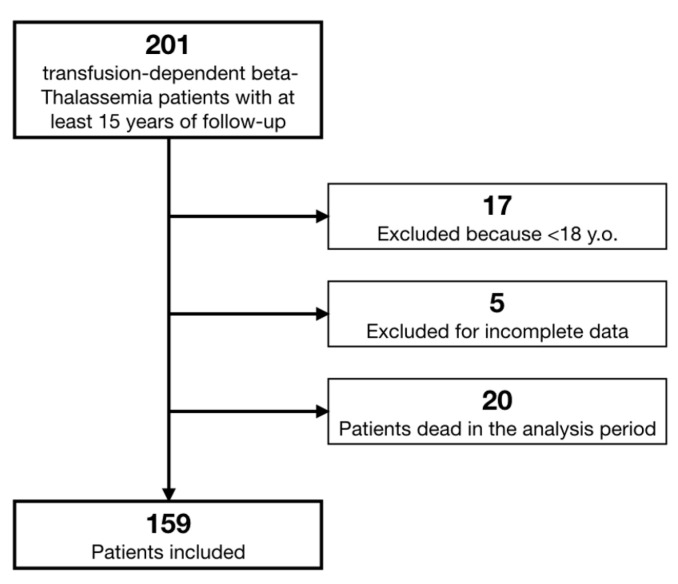
Study flow chart.

We retrieved data on 201 β-TM patients. According to pre-specified criteria, we excluded 18 patients because they were affected by Thalassemia Intermedia, 17 patients because they were younger than 18 years old, and 5 patients whose data were not sufficiently complete (Figure 1). Moreover, we registered a total mortality of 10% in the last 15 years, with 20 patients dead (9 of whom suffered a cardiac death).

Finally, 159 patients were included in our analysis: 82 females (52%) and 77 males (48%). The mean age was 40.9 ± 8.4 years, without statistical differences between males (40.3 ± 8.5) and females (41.4 ± 8.4; *p* = 0.395). All patients received an early diagnosis of TM (mean age at diagnosis 0.97 ± 0.94 years old) with prompt transfusion therapy (mean age at start 1.37 ± 1.49 years old), while the mean age at first chelation was 4.8 ± 3.1 years old, again without differences between males and females. Other clinical characteristics are shown in Table 1.

The prevalence of cardiovascular risk factors and other comorbidities are displayed in Table 2. A hypertension prevalence of 12% was found, with 19 patients on drug treatment. In addition, low serum lipid levels with low HDL levels were usually noted (49.6% of patients had HDL < 40 mg/dL), and 28% of patients had diabetes mellitus.

Sixty-two per cent of the population of this study had been diagnosed with hypogonadism, more frequently among women (69.5% vs. 53.2%, *p* = 0.03). Splenectomies were extremely frequent (111 people, 70% of the entire population), with a mean age at surgery of 17.3 ± 12 years and without differences among males and females (*p* = 0.4). The age at splenectomy was lower in patients with hypogonadism (9.5 ± 3.9 vs. 12.3 ± 6.1, *p* = 0.005). All 159 of the patients in the study were under iron chelating therapy at the time of this study (see Table 3). One hundred and thirty-seven patients (86%) changed the chelating treatment at least once. Iron-related parameters are shown in Table 4.

**Figure 2:**
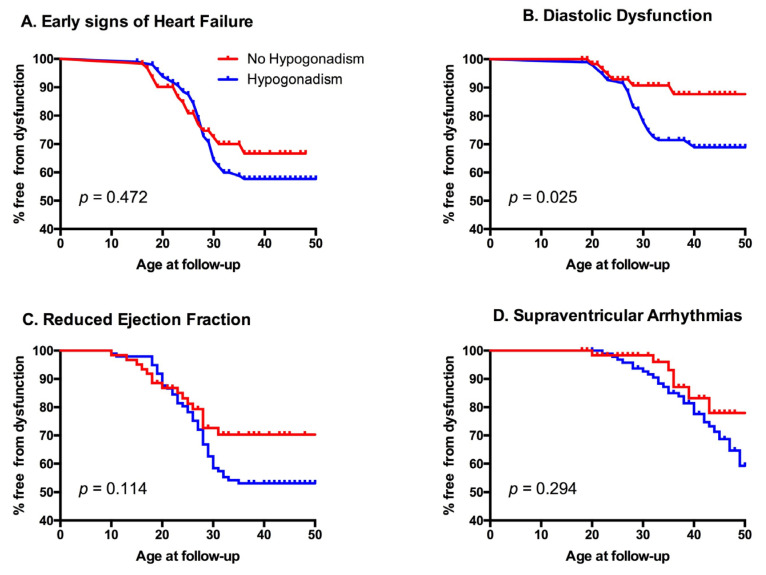
Kaplan–Meyer graphs of different cardiovascular outcomes according to gonadal state. Panel A: impact of hypogonadism on first diagnosis of early signs of heart failure; Panel B: impact of hypogonadism on the appearance of diastolic dysfunction; Panel C: impact of hypogonadism on first recognition of reduced ejection fraction; Panel D: impact of hypogonadism on supraventricular arrhythmias.

During the observed period, 6 patients (3.8%) had at least one episode of heart failure, 56 (35.9%) showed early signs of heart failure, 34 (22%) received a diagnosis of diastolic dysfunction, 60 (38%) developed a left ventricular ejection fraction <55%, and 33 (21.4%) had supraventricular arrhythmias (Table 5). Interestingly, patients with hypogonadism showed an increased rate of cardiovascular events (Figure 2). Cardiovascular risk was then assessed using two algorithms (CUORE and Pooled Cohort Risk Equation (PCRE)) and was generally low. The CUORE score showed mean values of 1.14 ± 1.25%, with a statistical difference between males and females (1.65 ± 1.57 vs. 0.66 ± 0.52 *p* < 0.001). ASCVD score showed mean values of 2.21 ± 2.3%, again with a statistical difference between the two genders (3.05 ± 2.74 vs. 1.43 ± 1.4 *p* < 0.001). Interestingly, patients affected by hypogonadism showed a peculiar metabolic profile, statistically different from the other patients (Table 6). No relationship was noted between the presence of metabolic syndrome and the other endocrinological diseases.

## 4. Discussion

Cardiomyopathy is a well-known complication in β-TM patients’ clinical history. However, despite the fact that cardiac iron overload has been believed to be the main actor in its progression for a long time, the main result of our study shows strong evidence that β-TM patients show a specific metabolic risk profile in which hypogonadism, one of the most common and well-known comorbidities in thalassemia patients [15,16], seems to play a peculiar and relevant role. A point of strength of our study is that the mean follow-up on chelation therapy was about 35 years, a span which covers the influence of all available drugs.

The high prevalence of this endocrine disease in β-TM patients is well known, usually presenting as hypogonadotropic hypogonadism due to the hemosiderosis of the gonadotroph cells, which is directly connected to the iron-overload severity and rarely reversible [17,18]. It may have a strong influence on the development of metabolic syndrome, altering hormonal levels [19]; previous studies have shown how hypogonadism might be associated with more severe cardiac siderosis, representing not only a risk factor for but also a marker of established cardiac disease [12,15].

Our results show a strong correlation between hypogonadism and pathologic cardiovascular outcomes, as shown in Table 7 and Figure 2 (Survival Tables are available as Supplementary Materials). Previous studies have shown that there is an increased occurrence of heart failure, metabolic syndrome, insulin resistance, and diabetes mellitus in hypogonadal patients [20]. Furthermore, gonadal dysfunction may interfere in cholesterol levels [19]. In our study these patients showed lower cardiac T2*, and had a statistically significant higher prevalence of diabetes mellitus and higher levels of total cholesterol and LDL. Interestingly, they also showed higher hepatic T2* values and higher liver stiffness as measured by FibroScan (i.e., transient liver elastography).

The role of hypogonadism might even be more complex. Intriguingly, its distribution in our cohort fits that of splenectomy history, with the mean age at splenectomy being seven years earlier than the age at first diagnosis of hypogonadism. Elderly patients, who more frequently had total splenectomies, may also have had inadequate chelation at a young age, both factors enhanced the hypophysis iron deposition, which in turn contributed to several hormonal impairments [21]. On top of that, splenectomies have a well-known role in increasing susceptibility to infections and the risk of developing pulmonary hypertension [1]; Further studies are needed to better clarify if the absence of a spleen is directly connected to the hypophysis iron deposition, but our data might represent a red flag for follow-up with this kind of patient, even if, by now, the traditional cardiovascular risk scores may appear low [22]. These considerations are important, since there are many reports of a safe and effective sex hormone replacement therapy in this population [23,24,25] but also because—at least in men—hormonal therapy could also induce adverse events and impact coronary plaque volume [23,26].

On the traditional assessment of cardiovascular risk, our cohort of β-TM patients presented a low prevalence of hypertension, but a high frequency of diabetes mellitus and smoking habits. In addition, the low levels of total cholesterol were actually hindered by a high prevalence of dyslipidemia with low values of HDL. For comparison, Italian data in the general 40-year-old population shows a diabetes prevalence below 2.5% and hypertension rate of less than 5%, with around 50% of people having a total cholesterol > 200 mg/dL. Smoking history in the general population at this age is around 40% (with 25% active smokers) [27,28]. In an attempt to predict the cardiovascular risk among our patients, we tried to apply two traditional risk scores, the CUORE [29] and the ASCVD [30]. Remarkably, these scores anticipated a low CVD risk. We think that the main reason for these results is that the traditional risk scores were derived from large group of people not including thalassemia major patients or intended for people older than fifty years old, and, therefore, these risk scores do not apply to β-TM patients. The most evident fallacy is that as of today β-TM patients still is a young population; in our cohort, only 18 (11.3%) were older than 50 years. The risk charts for cardiovascular events consider age as a very relevant factor, with an increased CVD risk seen in patients over 55 years of age [31]. Despite this, the life expectancy of β-TM patients has begun to increase with the introduction of iron chelation treatment in 1970. By now, the number of patients with β-thalassemia major reaching 55 years of age is still low, but it is growing [3,10]. On top of natural aging, pieces of evidence showing altered vascular aging in β-TM major patients have accumulated. Hahalis et al. showed impairment of arterial vasorelaxation and premature carotid atherosclerosis in β-TM patients [32], while Aggeli et al. showed that β-TM is associated with impaired endothelial function and increased levels of inflammatory cytokines [5]. Activation of vascular endothelium is considered an essential aspect of inflammation, vasculitis, and thrombosis, which, in turn, may enhance a prothrombotic state and accelerate vascular ageing, thus increasing the probability to develop atherosclerosis and coronary syndrome [33]. Finally, the relevant number of supraventricular arrhythmias reported in our population may be a clinical reflection of a diffuse pattern of iron-related myocardial fibrosis, which acts as a substrate for arrhythmia, which in turn, with the increasing age of patients, may become a significant clinical problem in terms of therapy and drug interaction [34]. β-TM populations appear to be facing a perfect storm of aging, endocrinological disorders, and metabolic syndrome that traditional risk scores cannot properly assess.

## 5. Conclusions

The chronic accumulation of iron in the heart and the specific metabolic profile, mainly observed in patients with hypogonadism, allows us to define Beta-Thalassemia Major as a high cardiovascular-risk disease from many points of view (iron-related myopathy, atherosclerosis risk factors, cardiovascular inflammations, and fibrosis-related arrhythmias) that, taking into account the increasing life expectancy of these patients, requires a specific follow-up program to identify early signs and prevent serious complications.

## Figures and Tables

**Table 1:** Population clinical characteristics.

	Total (*n* = 159)	Male (*n* = 77)	Female (*n* = 82)	*p*-Value
Age (years)	40.9 ± 8.4	40.3 ± 8.5	41.4 ± 4.4	0.4
Diagnosis (years)	0.97 ± 0.94	1.03 ± 0.98	0.91 ± 0.9	0.4
First transfusion (years)	1.37 ± 1.49	1.33 ± 1.23	1.41 ± 1.71	0.7
Chelation start (years)	4.8 ± 3.1	4.45 ± 2.74	5.13 ± 3.32	0.17
Height (cm)	160.5 ± 9.1	165.9 ± 7.3	155.3 ± 7.5	<0.001
Weight (Kg)	59.7 ± 11.8	65 ± 11.7	54.7 ± 9.7	<0.001
BMI (Kg/m^2^)	23. ± 3.4	23.5 ± 3.6	22.6 ± 3.1	0.08
Body surface area (m^2^)	1.62 ± 0.19	1.72 ± 0.17	1.52 ± 0.16	<0.001
Heart rate (bpm)	81.7 ± 8.2	81.6 ± 9.2	81.8 ± 7.3	0.86

BMI: Body Mass Index (kg/m^2^)

**Table 2:** Cardiovascular risk factors and comorbidities.

	Total (*n* = 159)	Male (*n* = 77)	Female (*n* = 82)	*p*-Value
Smoke history	52 (32.7%)	27 (35.1%)	25 (30.5%)	0.33
Hypertensive (mmHg)	19 (12%)	13 (16.9%)	6 (7.3%)	0.053
Dyslipidemia	45 (28%)	22 (30%)	23 (29.5%)	0.54
Total cholesterol (mg/dL)	142.6 ± 36.6	133.9 ± 38.6	150.8 ± 32.7	0.004
HDL (mg/dL)	40.9 ± 3.7	37.2 ± 12.8	44.3 ± 13.7	0.001
LDL (mg/dL)	73.2 ± 32.4	65.9 ± 32.6	80.2 ± 30.8	0.005
Triglycerides (mg/dL)	125.7 ± 73.7	135.7 ± 78.7	116.2 ± 67.8	0.07
Hypothyroidism	37 (23.3%)	15 (19.5%)	22 (26.8%)	0.182
Hypoparathyroidism	9 (5.7%)	7 (9.1%)	2 (2.4%)	0.07
Hypogonadism	98 (61.6%)	41 (53.2%)	57 (69.5%)	0.03
Diabetes mellitus	28 (17.6%)	11 (14.3%)	17 (20.7%)	0.2
Altered Glucose Tolerance	15 (11.5%)	5 (7.6%)	10 (15.4%)	0.13
Impaired Fasting Glucose	4 (3.1%)	2 (2.8%)	2 (2.4%)	0.67
Hyperinsulinism	19 (14.5%)	7 (106%)	12 (18.5%)	0.15
Splenectomy	111 (69.8%)	55 (71.4%)	56 (68.3%)	0.4

HDL: High Density Lipoprotein; LDL: Low Density Lipoprotein

**Table 3:** Distribution of iron chelation therapy.

	N	Deferoxamine	Deferasirox	Deferiprone
Now				
Single	134 (84%)	19	83	32
Combined	25 (16%)	13	15	22
Total	159 (100%)	22	98	54

**Table 4:** Iron related parameters.

	Mean	Male (*n* = 77)	Female (*n* = 82)	*p*-Value
Ferritin (last year) (ng)	1671 ± 1577	1799 ± 1597	1551 ± 1559	0.3
Ferritin (five years) (ng)	1636 ± 1591	1652 ± 1361	1622 ± 1788	0.9
Iron input *	8706 ± 2232	9467 ± 2465	7992 ± 1717	<0.001
T2* Heart (ms)	34.65 ± 10.17	35.06 ± 9.76	34.27 ± 10.59	0.6
T2* Liver (ms)	10.90 ± 6.65	10.49 ± 6.23	11.27 ± 7.05	0.5

* Iron input is the estimate of the iron intake within blood transfusion, measured in mg. T2*: T2*-weighted imaging is an MRI sequence to quantify observable or effective T2. In this sequence, hemorrhages and hemosiderin deposits become hypointense

**Table 5:** Cardiovascular outcomes distribution.

	Total	Age (Years)	Male	Age (Years)	Female	Age (Years)	*p*-Value
Heart Failure	6 (3.8%)	26.5 ± 3.2	3	26. ± 2.0	3	27 ± 4.6	0.70
Early signs of HF	57 (35.9%)	25.8 ± 5.0	31	25 ± 4.8	26	27 ± 5.0	0.17
Diastolic dysfunction	35 (22%)	28.5 ± 6.5	20	27.9 ± 5.6	15	28 ± 7.7	0.16
LVEF < 55%	61 (38%)	23.9 ± 5.9	34	23.6 ± 6.3	27	24.3 ± 5.4	0.09
Arrhythmias	34 (21.4%)	35.4 ± 7.2	17	37 ± 8	17	39 ± 8.0	0.12

HF: Heart Failure; LVEF: Left Ventricular Ejection Fraction.

**Table 6:** Clinical characteristics of patients with and without hypogonadism.

	With Hypogonadism		Without Hypogonadism		
	N	Mean	N	Mean	*p*-Value
Male (%)	42%		59%		0.03
Female (%)	58%		41%		0.03
Age (years)	98	44 ± 7	61	36 ± 9	<0.001
First transfusion (years)	98	1.3 ± 1.5	61	1.5 ± 1.4	0.3
First chelation (years)	98	5.2 ± 3	61	4.1 ± 2.6	0.03
T2* heart (ms)	96	32 ± 10	61	39 ± 9	<0.001
T2* liver (ms)	96	12 ± 7	61	9 ± 6	0.01
Liver stiffness (kPa)	98	6.53 ± 2.4	60	5.72 ± 1.9	0.03
Total cholesterol (mg/dL)	96	142 ± 36	55	134 ± 34	0.04
HDL (mg/dL)	96	43 ± 14	55	38 ± 12	0.03
LDL (mg/dL)	96	81 ± 31	55	61 ± 30	<0.001
Diabetes mellitus	24.5%		6.6%		0.003
Splenectomy	80%		54%		0.001
HCVab	82.7%		49.2%		<0.001

T2*: T2*-weighted imaging is an MRI sequence to quantify observable or effective T2; HDL: High Density Lipoprotein; LDL: Low Density Lipoprotein; HCVab: HCV antibodies.

**Table 7:** Cardiovascular outcomes and hypogonadism.

	With Hypogonadism (*n* = 98)	Without Hypogonadism (*n* = 61)	*p*-Value
Early signs of HF	40 (41%)	17 (28%)	0.09
Diastolic dysfunction	29 (30%)	6 (10%)	0.03
LVEF < 55%	45 (46%)	16 (26%)	0.01
Arrhythmias	27 (28%)	7 (12%)	<0.001

HF: Heart Failure; LVEF: Left Ventricular Ejection Fraction.

## Data Availability

Data are available on personal request to the corresponding author.

## Supplementary Materials

The following are available online at https://www.mdpi.com/article/10.3390/jcdd9010003/s1. Table S1: Survival Table 1: early signs of heart failure; Table S2: Survival Table 2: Diastolic dysfunction; Table S3: Survival Table 3: Ejection fraction below 55%; Table S4: Survival Table 4: Supraventricular arrhythmias.

## References

[1] Pennell D.J., Udelson J.E., Arai A.E., Bozkurt B., Cohen A.R., Galanello R., Hoffman T.M., Kiernan M.S., Lerakis S., Piga A. (2013). Cardiovascular function and treatment in β-thalassemia major: A consensus statement from the American Heart Association. Circulation.

[2] Galanello R., Origa R. (2010). Beta-thalassemia. Orphanet. J. Rare Dis..

[3] Longo F., Corrieri P., Origa R., Barella S., Sanna P.M.G., Bitti P.P., Zuccarelli A., Commendatore F.V., Vitucci A., Quarta A. (2020). Changing patterns of thalassaemia in Italy: A WebThal perspective. Blood Transfus. Trasfus. Sangue..

[4] Betts M., Flight P.A., Paramore L.C., Tian L., Milenković D., Sheth S. (2020). Systematic literature review of the burden of disease and treatment for transfusion-dependent β-Thalassemia. Clin. Ther..

[5] Aggeli C., Antoniades C., Cosma C., Chrysohoou C., Tousoulis D., Ladis V., Karageorga M., Pitsavos C., Stefanadis C. (2005). Endothelial dysfunction and inflammatory process in transfusion-dependent patients with beta-thalassemia major. Int. J. Cardiol..

[6] Gullu H., Caliskan M., Caliskan Z., Unler G.K., Ermisler E., Ciftci A.G., Guven A., Muderrisoglu H. (2013). Coronary microvascular function, peripheral endothelial function and carotid IMT in beta-thalassemia minor. Thromb. Res..

[7] Sherief L.M., Dawood O., Ali A., Sherbiny H.S., Kamal N.M., Elshanshory M., Alazez O.A., Alhady M.A., Nour M., Mokhtar W.A. (2017). Premature atherosclerosis in children with beta-thalassemia major: New diagnostic marker. BMC Pediatr..

[8] Barbero U., Destefanis P., Pozzi R., Longo F., Piga A. (2012). Exercise stress echocardiography with tissue doppler imaging (TDI) detects early systolic dysfunction in beta-thalassemia major patients without cardiac iron overload. Mediterr. J. Hematol. Infect. Dis..

[9] Barbero U., Longo F., Destefanis P., Gaglioti C.M., Pozzi R., Piga A. (2016). Worsening of myocardial performance index in beta-thalassemia patients despite permanently normal iron load at MRI: A simple and cheap index reflecting cardiovascular involvement?. IJC Metab. Endocr..

[10] Farmakis D., Giakoumis A., Angastiniotis M., Eleftheriou A. (2020). The changing epidemiology of the ageing thalassaemia populations: A position statement of the Thalassaemia International Federation. Eur. J. Haematol..

[11] Voskaridou E., Kattamis A., Fragodimitri C., Kourakli A., Chalkia P., Diamantidis M., Vlachaki E., Drosou M., Lafioniatis S., on behalf of the Greek Haemoglobinopathies Study Group (2018). National registry of hemoglobinopathies in Greece: updated demographics, current trends in affected births, and causes of mortality. Ann. Hematol..

[12] Ehsan L., Rashid M., Alvi N., Awais K., Nadeem O., Asghar A., Sajjad F., Fatima M., Qidwai A., Hussain S. (2018). Clinical utility of endocrine markers predicting myocardial siderosis in transfusion dependent thalassemia major. Pediatr. Blood Cancer.

[13] Lang R.M., Bierig M., Devereux R.B., Flachskampf F.A., Foster E., Pellikka P.A., Picard M., Roman M.J., Seward J., Shanewise J.S. (2005). Recommendations for chamber quantification: A report from the American Society of Echocardiography’s Guidelines and Standards Committee and the Chamber Quantification Writing Group, developed in conjunction with the European Association of Echocardiography, a branch of the European Society of Cardiology. J. Am. Soc. Echocardiogr..

[14] Fonarow G.C., Scientific Advisory Committee (2003). The acute decompensated heart Failure national registry: Opportunities to improve care of patients hospitalized with acute decompensated heart failure. Rev. Cardiovasc. Med..

[15] Ang A.L., Tzoulis P., Prescott E., Davis B.A., Barnard M., Shah F.T. (2014). History of myocardial iron loading is a strong risk factor for diabetes mellitus and hypogonadism in adults with β thalassemia major. Eur. J. Haematol..

[16] De Sanctis V., Soliman A.T., A Yassin M., Di Maio S., Daar S., Elsedfy H., Soliman N., Kattamis C. (2018). Hypogonadism in male thalassemia major patients: pathophysiology, diagnosis and treatment. Acta Biomed.

[17] Srisukh S., Ongphiphadhanakul B., Bunnag P. (2016). Hypogonadism in thalassemia major patients. J. Clin. Transl. Endocrinol..

[18] De Sanctis V., Soliman A., Elsedfy H., Di Maio S., Canatan D., Soliman N., Karimi M., Kattamis C. (2017). Gonadal dysfunction in adult male patients with thalassemia major: an update for clinicians caring for thalassemia. Expert Rev. Hematol..

[19] Talaulikar V.S., Bajoria R., Ehidiamhen A.J., Mujawar E., Chatterjee R. (2019). A 10-year longitudinal study of evaluation of ovarian reserve in women with transfusion-dependent beta thalassaemia major. Eur. J. Obstet. Gynecol. Reprod. Biol..

[20] Auerbach J.M., Khera M. (2020). Hypogonadism management and cardiovascular health. Postgrad. Med..

[21] Moshtaghi-Kashanian G.-R., Razavi F. (2009). Ghrelin and leptin levels in relation to puberty and reproductive function in patients with beta-thalassemia. Hormones.

[22] Cappellini M.D., Viprakasit V., Taher A.T., Georgiev P., Kuo K.H., Coates T., Voskaridou E., Liew H.-K., Pazgal-Kobrowski I., Forni G. (2020). A Phase 3 Trial of Luspatercept in Patients with Transfusion-Dependent β-Thalassemia. N. Engl. J. Med..

[23] De Sanctis V., Soliman A.T., Daar S., Di Maio S. (2019). Adverse events during testosterone replacement therapy in 95 young hypogonadal thalassemic men. Acta Bio-Medica. Atenei. Parm..

[24] De Sanctis V., Soliman A.T., Daar S., Di Maio S., Yassin M.A., Canatan D., Corrons J.-L.V., Elsedfy H., Kattamis A., Kattamis C. (2019). The experience of a tertiary unit on the clinical phenotype and management of hypogonadism in female adolescents and young adults with transfusion dependent thalassemia. Acta Bio-Medica. Atenei. Parm..

[25] De Sanctis V., Soliman A.T., Elsedfy H., Albu A., Al Jaouni S., Anastasi S., Bisconte M.G., Canatan D., Christou S., Daar S. (2017). Review and recommendations on management of adult female thalassemia patieνts with hypogonadism based on literature review and experience of ICET-A network specialists. Mediterr. J. Hematol. Infect. Dis..

[26] Budoff M.J., Ellenberg S.S., Lewis C.E., Mohler E.R., Wenger N.K., Bhasin S., Barrett-Connor E., Swerdloff R.S., Stephens-Shields A., Cauley J.A. (2017). Testosterone treatment and coronary artery plaque volume in older men with low testosterone. JAMA.

[27] Marzetti E., Calvani R., Picca A., Sisto A., Tosato M., Martone A.M., Ortolani E., Salini S., Pafundi T., Santoliquido A. (2018). Prevalence of dyslipidaemia and awareness of blood cholesterol levels among community-living people: results from the Longevity check-up 7+ (Lookup 7+) cross-sectional survey. BMJ Open.

[28] Istituto Superiore di Sanità. Sistema di Sorveglianza PASSI (Progressi delle Aziende Sanitarie per la Salute in Italia) (2007). Gruppo Tecnico di Coordinamento del Progetto di Sperimentazione del “Sistema di Sorveglianza PASSI”.

[29] Palmieri L., Panico S., Vanuzzo D., Ferrario M., Pilotto L., Sega R., Cesana G. (2004). La valutazione del rischio cardiovascolare globale assoluto: Il punteggio individuale del Progetto CUORE. Ann. Ist. Super Sanita..

[30] Goff D.C., Lloyd-Jones D.M., Bennett G., Coady S., D’Agostino R.B., Gibbons R., Greenland P., Lackland D.T., Levy D., O’Donnell C.J. (2014). 2013 ACC/AHA guideline on the assessment of cardiovascular risk: A report of the American College of Cardiology/American Heart Association Task Force on practice guidelines. Circulation.

[31] Visseren F.L.J., Mach F., Smulders Y.M., Carballo D., Koskinas K.C., Bäck M., Benetos A., Biffi A., Boavida J.-M., Capodanno D. (2021). 2021 ESC Guidelines on cardiovascular disease prevention in clinical practice. Eur. Heart J..

[32] Hahalis G., Kremastinos D.T., Terzis G., Kalogeropoulos A.P., Chrysanthopoulou A., Karakantza M., Kourakli A., Adamopoulos S., Tselepis A.D., Grapsas N. (2008). Global vasomotor dysfunction and accelerated vascular aging in β-thalassemia major. Atherosclerosis.

[33] Barbero U., D’Ascenzo F., Nijhoff F., Moretti C., Biondi-Zoccai G., Mennuni M., Capodanno D., Lococo M., Lipinski M.J., Gaita F. (2016). Assessing risk in patients with stable coronary disease: When should we intensify care and follow-up? Results from a meta-analysis of observational studies of the courage and fame era. Scientifica.

[34] Barbero U., Fornari F., Guarguagli S., Gaglioti C.M., Longo F., Doronzo B., Anselmino M., Piga A. (2018). Atrial fibrillation in β-thalassemia major patients: Diagnosis, management and therapeutic options. Hemoglobin.

