# Supplementary material for: The Influence of Cardiovascular Risk Factors and Hypogonadism on Cardiac Outcomes in an Aging Population of Beta-Thalassemia Patients
Source: J Cardiovasc Dev Dis. 2021 Dec 21;9(1):3. doi: 10.3390/jcdd9010003 (PMC8781644; doi:10.3390/jcdd9010003)
Supplement: Supplementary file 1 [file jcdd-09-00003-s001.zip › jcdd-1441055-supplementary.pdf]

Table S1. Survival Table 1: early signs of heart failure.

First Order Controls		Interval Start Time	Number Entering Interval	Number Withdrawing during Interval	Exposed to Risk	Number of Terminal Events	Proportion Terminating	Proportion Surviving	Cumulative Proportion Surviving at the End of Interval	Standard Error	Probability density	Standard Error	Hazard Rate	Standard Error
		0	61	0	61.000	0	0.00	1.00	1.00	0.00	0.000	0.000	0.00	0.00
		10	61	2	60.000	6	0.10	0.90	0.90	0.04	0.010	0.004	0.01	0.00
	0	20	53	13	46.500	8	0.17	0.83	0.75	0.06	0.015	0.005	0.02	0.01
		30	32	16	24.000	3	0.13	0.88	0.65	0.07	0.009	0.005	0.01	0.01
Hypogonadism		40	13	13	6.500	0	0.00	1.00	0.65	0.07	0.000	0.000	0.00	0.00
		0	98	0	98.000	0	0.00	1.00	1.00	0.00	0.000	0.000	0.00	0.00
		10	98	2	97.000	4	0.04	0.96	0.96	0.02	0.004	0.002	0.00	0.00
	1	20	92	2	91.000	24	0.26	0.74	0.71	0.05	0.025	0.004	0.03	0.01
		30	66	8	62.000	12	0.19	0.81	0.57	0.05	0.014	0.004	0.02	0.01
		40	46	34	29.000	0	0.00	1.00	0.57	0.05	0.000	0.000	0.00	0.00
			12	12	6.000	0	0.00	1.00	0.57	0.05	0.000	0.000	0.00	0.00

Table S2. Survival Table 2: Diastolic dysfunction.

First Order Controls	Interval Start Time	Number Entering Interval	Number Withdrawing during Interval	Exposed to Risk	Number of Terminal Events	Proportion Terminating	Proportion Surviving	Cumulative Proportion Surviving at the End of Interval	Standard error	Probability Density	Standard Error	Hazard Rate	Standard Error
	0	61	0	61.000	0	0.00	1.00	1.00	0.00	0.000	0.000	0.00	0.00
	10	61	2	60.000	0	0.00	1.00	1.00	0.00	0.000	0.000	0.00	0.00
0	20	59	14	52.000	5	0.10	0.90	0.90	0.04	0.010	0.004	0.01	0.00
	30	40	21	29.500	1	0.03	0.97	0.87	0.05	0.003	0.003	0.00	0.00
	40	18	17	9.500	0	0.00	1.00	0.87	0.05	0.000	0.000	0.00	0.00
Hypogonadism	50	1	1	0.500	0	0.00	1.00	0.87	0.05	0.000	0.000	0.00	0.00
	0	98	0	98.000	0	0.00	1.00	1.00	0.00	0.000	0.000	0.00	0.00
1	10	98	2	97.000	1	0.01	0.99	0.99	0.01	0.001	0.001	0.00	0.00
	20	95	2	94.000	16	0.17	0.83	0.82	0.04	0.017	0.004	0.02	0.00
	30	77	11	71.500	11	0.15	0.85	0.69	0.05	0.013	0.004	0.02	0.01
	40	55	41	34.500	1	0.03	0.97	0.67	0.05	0.002	0.002	0.00	0.00
	50	13	13	6.500	0	0.00	1.00	0.67	0.05	0.000	0.000	0.00	0.00

Table S3. Survival Table 3: Ejection fraction below 55%.

First Order Controls		Interval Start Time	Number Entering Interval	Number Withdrawing during Interval	Exposed to Risk	Number of Terminal Events	Proportion Terminating	Proportion Surviving	Cumulative Proportion Surviving at the End of Interval	Standard Error	Probability Density	Standard Error	Hazard Rate	Standard Error
		0	61	0	61.000	0	0.00	1.00	1.00	0.00	0.000	0.000	0.00	0.00
		10	61	2	60.000	7	0.12	0.88	0.88	0.04	0.012	0.004	0.01	0.00
	0	20	52	13	45.500	8	0.18	0.82	0.73	0.06	0.016	0.005	0.02	0.01
		30	31	16	23.000	1	0.04	0.96	0.70	0.07	0.003	0.003	0.00	0.00
		40	14	12	8.000	0	0.00	1.00	0.70	0.07	0.000	0.000	0.00	0.00
Hypogonadism		50	2	2	1.000	0	0.00	1.00	0.70	0.07	0.000	0.000	0.00	0.00
		0	98	0	98.000	0	0.00	1.00	1.00	0.00	0.000	0.000	0.00	0.00
		10	98	1	97.500	8	0.08	0.92	0.92	0.03	0.008	0.003	0.01	0.00
	1	20	89	1	88.500	28	0.32	0.68	0.63	0.05	0.029	0.005	0.04	0.01
		30	60	5	57.500	9	0.16	0.84	0.53	0.05	0.010	0.003	0.02	0.01
		40	46	34	29.000	0	0.00	1.00	0.53	0.05	0.000	0.000	0.00	0.00
		50	12	12	6.000	0	0.00	1.00	0.53	0.05	0.000	0.000	0.00	0.00

Table S4. Survival Table 4: Supraventricular arrhythmias**.**

First Order Controls		Interval Start Time	Number Entering Interval	Number Withdrawing during Interval	Exposed to Risk	Number of Terminal Events	Proportion Terminating	Proportion Surviving	Cumulative Proportion Surviving at the End of Interval	Standard Error	Probability Density	Standard Error	Hazard Rate	Standard Error
		0	61	0	61.000	0	0.00	1.00	1.00	0.00	0.000	0.000	0.00	0.00
		10	61	2	60.000	0	0.00	1.00	1.00	0.00	0.000	0.000	0.00	0.00
	0	20	59	13	52.500	1	0.02	0.98	0.98	0.02	0.002	0.002	0.00	0.00
		30	45	21	34.500	5	0.14	0.86	0.84	0.06	0.014	0.006	0.02	0.01
Hypogonadism		40	19	17	10.500	1	0.10	0.90	0.76	0.09	0.008	0.008	0.01	0.01
		50	1	1	0.500	0	0.00	1.00	0.76	0.09	0.000	0.000	0.00	0.00
		0	98	0	98.000	0	0.00	1.00	1.00	0.00	0.000	0.000	0.00	0.00
		10	98	2	97.000	0	0.00	1.00	1.00	0.00	0.000	0.000	0.00	0.00
	1	20	96	2	95.000	6	0.06	0.94	0.94	0.02	0.006	0.002	0.01	0.00
		30	88	14	81.000	11	0.14	0.86	0.81	0.04	0.013	0.004	0.01	0.00
		40	63	46	40.000	10	0.25	0.75	0.61	0.06	0.020	0.006	0.03	0.01
		50	7	7	3.500	0	0.00	1.00	0.61	0.06	0.000	0.000	0.00	0.00
